# Methylphenidate or Family Mindfulness? Effects on Internalizing, Externalizing and Social Problems in Children with Attention Deficit Hyperactivity Disorder

**DOI:** 10.3390/children12060681

**Published:** 2025-05-26

**Authors:** Brett Kosterman Zoller, Susan M. Bögels, Renee Meppelink, Esther I. de Bruin

**Affiliations:** 1 Research Institute of Child Development and Education (RICDE), University of Amsterdam, 1018 WS Amsterdam, The Netherlands; b.d.kosterman@uva.nl; 2 Developmental Psychology, University of Amsterdam, 1018 WB Amsterdam, The Netherlands; s.m.bogels@uva.nl; 3 Arkin Youth and Family, 1058 AA Amsterdam, The Netherlands; renee.meppelink@arkinjeugd.nl; 4 UvA Minds, Academic Treatment Centre, University of Amsterdam, 1071 JW Amsterdam, The Netherlands

**Keywords:** family-based mindfulness, MYmind, methylphenidate, externalizing problems, internalizing problems, social problems, ADHD

## Abstract

Background: Externalizing, internalizing and social problems are frequent comorbidities for children with ADHD. This study explored the effects of methylphenidate versus a child and parents’ parallel mindfulness program (MYmind) on children’s internalizing, externalizing and social problems. Data came from our RCT investigating treatment effects on the primary outcome of children’s ADHD symptoms. Methods: Children followed their ascribed course of treatment for 4 months: 46 children were in the mindfulness group and 42 in the medication group. Multilevel modeling analyzed the responses of four informants (mothers, fathers, children and teachers) across three follow-up points: short-term (2 months), medium-term (4 months) and long-term (10 months). Results: Both treatment groups improved from the baseline across all outcomes. No differences were found between groups on improvement of mindful awareness as reported by children at any time point. At the short-term follow-up, children in the medication group showed greater reductions as compared to those in the mindfulness group on internalizing problems (as observed by mothers), externalizing problems (mothers; fathers) and social problems (mothers; teachers). At the medium-term follow-up, children in the medication group still showed larger reductions in externalizing problems as observed by fathers, but on all other outcomes, informants’ reports did not differ between groups. At the long-term follow-up, none of the informants reported any differences between treatment groups in effects on internalizing, externalizing or social problems. Conclusions: Given the observations of similar improvements at short-, medium- and long-term, mindfulness might be a viable option for families of children with ADHD who are seeking an alternative to medication to reduce comorbid externalizing, internalizing and/or social problems.

## 1. Introduction

Attention deficit hyperactivity disorder (ADHD) is a neurodevelopmental disorder that is estimated to affect between 3.5% and 11% of children [1,2,3]. It is characterized by problems with inattention, hyperactivity and impulsivity, which can lead to impairment in functioning in daily life [4,5]. There is an increased risk of psychiatric comorbidities for children with ADHD [6], e.g., two out of three children with ADHD also meet criteria for at least one other clinical diagnosis [1,7,8].

Treatment can be complicated because problems are often exacerbated by comorbidity [9] and transdiagnostic traits (traits shared by disorders) [10,11], as well as the potential overlap of underlying causes of dysfunctional behavior, such as deficits in executive functioning and emotional regulation. Specifically, internalizing, externalizing and social problems are common comorbidities for children with ADHD [9,12].

Internalizing disorders are prevalent among children with ADHD: 15–30% present with anxiety disorders [1,8,13], 25% with depression [13] and 94% with somatic complaints [14]. ADHD is further significantly correlated to cyclothymic-related temperaments as stable traits, indicating the potential of moods swinging from high to low [15]. While the presence of anxiety can contribute to an increase in inhibition and work as a protective factor against impulsive behavior [16], it is also associated with more attention problems [13]. Depression is often increased in children with ADHD and can be a product of demoralization if children fall short of their peers’ performance [17] or experience increased peer rejection and family conflict. Depressive symptoms can also impact other factors that are known to mediate ADHD characteristics, such as psychosocial functioning [18] and executive functioning [19]. The presence of anxiety might strengthen the interaction between ADHD and levels of externalizing behaviors [20], indicating an interaction between presenting problems.

Externalizing disorders affect between 30% and 50% of children with ADHD [1] and manifest primarily as oppositional defiant disorder (ODD) and/or conduct disorder (CD), affecting roughly 20% [7] and 33% [21] of children, respectively. ODD can be similar to ADHD in how it is experienced by those around the child. While a comparable level of impulsivity may manifest as disruptive, externalizing behavior, there may be different root mechanisms of impulsivity at play in ODD versus ADHD [22]. Similarly, both CD and ADHD may manifest in common negative outcomes such as criminality or substance abuse, but the root mechanisms are likely to be different [23]. It is also found that untreated ADHD is associated with worse academic performance in the long term. However, multimodal treatment improved the academic performance of these youngsters [24].

A third area that can negatively impact children with ADHD is social problems, including peer rejection, being bullied, feeling intimidated, isolation, loneliness and over-dependence on parents [25]. One study found peer rejection as high as 52% for children with ADHD [26]. ADHD is associated with poorer social skills [27], which can exacerbate depression and reduce social support and interaction [28], contributing to a potential cycle, in that ADHD combined with depression can contribute to peer isolation [29]. ADHD inattention symptoms may increase anxiety due to poor academic performance [28], diminished interpersonal relationships [30,31], difficulties in the recognition of emotional facial cues that further impair relationships [32] and strains in parent–child relationships [30,33]. ADHD in children is also associated with reduced pro-social behavior [34] that can contribute to a toxic mix if comorbid conduct and ODD problems [35] increase hostility towards peers [36].

Methylphenidate is the most prescribed stimulant medication and is effective in reducing symptoms in children with ADHD [37]. While being prescribed exclusively for treating ADHD, studies have explored potential effects on children’s comorbid symptoms. A recent meta-study found that ADHD medication does not have an effect on anxiety and depression compared to placebo controls [38]. However, methylphenidate was found to reduce somatic symptoms [39]. Another study found that children using methylphenidate showed improved emotion regulation, which can, in turn, lead to lower levels of externalizing symptoms [40]. A meta-study also showed that methylphenidate was successful in reducing children’s social problems [41]. Despite its effectiveness, medication for children comes with several drawbacks such as insomnia, loss of appetite, anxiety, nervosity, stomachaches [37], symptoms return once medication is stopped (rebound effect) [42], treatment adherence is often low [43] and effectiveness in the long term is unclear [44].

Inspired and often modeled after Kabat-Zinn’s eastern meditation-based Mindfulness-Based Stress Reduction [45], mindfulness-based interventions (MBIs) have been found in RCTs to be as effective and even superior alternatives to other evidence-based psycho-social interventions in meta-studies across a myriad of targeted problems and multiple populations [46]. At their core, competent-instructor-led MBIs are trainings informed by contemplative practices (meditation), which focus on attention training to increase awareness and concentration, and reduce automatic responding [45] combined with knowledge from psychology, medicine and education [47].

Participants in the MYmind intervention learn to focus their attention on an anchor (e.g., body or breath), notice when their minds wander and then practice redirecting their attention. Children with ADHD suffer from difficulties paying or maintaining attention, which seems to match well with mindfulness-based programs since they essentially focus on attention training, where children (and their parents) learn to better control their attention, not only during the training but also through practices they can use in daily life. Children with ADHD often show hyperactive and impulsive reactions. During a mindfulness training, they practice becoming more aware of thoughts, bodily feelings, emotions and impulses. Instead of automatically responding or instantly reacting, an increased awareness and attention can help them inhibit their first responses and react more consciously. Parents, in turn, learn to become more patient and compassionate towards themselves and the behavior of their child, and the above-mentioned attention skills might be useful to them too, since they often recognize some of the ADHD behaviors in themselves.

The MYmind family-based mindfulness program has been shown to be effective in reducing children’s ADHD symptoms [48,49] and has been included in meta-analyses and systematic reviews, demonstrating the effectiveness of mindfulness-based interventions (MBIs) in children with ADHD [50,51]. In contrast to methylphenidate, MBIs can be used to treat many of the comorbidity symptoms associated with childhood ADHD, e.g., MBIs have been found to reduce internalizing and externalizing symptoms in children with ADHD [52,53]. While two meta-studies have found that mindfulness may not significantly improve children’s anxiety and depression symptoms [53,54,55], nor significantly reduce children’s aggressive behavior [56], it may be more promising for treating children’s internalizing and somatic symptoms [57].

The aim of the current study is to examine the effects of a family MBI compared to medication (methylphenidate; MPH) as one of the first lines of treatment for ADHD worldwide on children’s comorbid internalizing, externalizing and social problems. In our previous work using the same dataset, we found that medication was immediately effective in reducing children’s ADHD symptoms, while meditation took more time to become effective [48]. The novelty of the current study is the emphasis on the often underestimated but highly prevalent comorbid symptoms, which may cause these children and their families extra suffering. Given the potential underlying causes, interaction and mimicking of presenting symptoms of ADHD, internalizing, externalizing and social problems, we expect to find mindfulness and medication to also have modest effects on reducing children’s comorbid symptoms. We hypothesize that medication will produce more immediate effects than mindfulness.

## 2. Materials and Methods

### 2.1. Participants

Families were recruited via referrals from general practitioners, mental health care professionals, the website for the study (www.adhd-meditatieofmedicatie.nl), posters, flyers and local media outlets (including newspapers, radio, magazines and social media sources). Children were screened at either UvA minds or Buro van Roosmalen to verify diagnostic criteria and that they were in accordance with protocol exclusion criteria [58]. Families were randomly assigned to either child medication or family mindfulness training. In total, 377 families were assessed for eligibility, with 98 being randomly assigned to the RCT; 88 received the allocated treatment: 46 received mindfulness and 42 medication. Please see ref. [48] for participant characteristics and the CONSORT flowchart.

### 2.2. Measures

#### 2.2.1. Internalizing Problems

Internalizing, externalizing and social problems were measured using the Achenbach System of Empirically Based Assessment (ASEBA) [59]. The ASEBA is a series of tests on children’s behaviors. The ASEBA’s Child Behavior Checklist (CBCL, 113 items, parents’ report), Youth Self Report (YSR, 112 items, self-report for adolescents 11 years and older) and Teacher’s Report Form (TRF, 113 items, teacher report) were used. We utilized the ASEBA score of Internalizing Problems, which is composed of three subscales: Anxious/Depressed (AD), Withdrawn/Depressed (WD) and Somatic Complaints (SC). Examples from the subscales, as filled out by parents in the CBCL about their child, are “cries a lot”, “there is very little he/she enjoys” and “feels dizzy or lightheaded”, respectively. The parents’ CBCL consists of 32 items: AD = 13, WD = 8 and SC = 11. The adolescents’ YSR is composed of 31 items: AD = 13, WD = 8 and SC = 10. The Teacher’s TRF has 33 items: AD = 16, WD = 8 and SC = 9. Internal reliabilities at pretest, 2 months, 4 months and 10 months were CBCL: α = 0.85, 0.85, 0.82, 0.86; YSR: α = 0.86, 0.88, 0.91, 0.91; TRF: α = 0.83, 0.82 (teachers did not complete the 4-month or 10-month assessment).

#### 2.2.2. Externalizing Problems

Please see Internalizing Problems above for a description of the Achenbach system. The construct of Externalizing Problems is composed of two subscales: Rule-Breaking Behavior (RBB) and Aggressive Behavior (AB). Examples from the parents’ CBCL form are “drinks alcohol without parents’ approval” and “argues a lot”. For Externalizing Problems, the CBCL has 35 items: RBB = 17 and AB = 18. The YSR covers 32 items: RBB = 15 and AB = 17. And the TRF has 32 items: RBB = 12 and AB = 20. Internal reliabilities at pretest, 2 months, 4 months and 10 months were CBCL, α = 0.90, 0.92, 0.89, 0.90; YSR, α = 0.82, 0.88, 0.92, 0.93; TRF, α = 0.94, 0.94.

#### 2.2.3. Social Problems

Please see Internalizing Problems above for a description of the Achenbach system. Examples of measures for Social Problems from the CBCL are “clings to adults or too dependent” and “complains of loneliness”. All three respondent measurements (CBCL, YSR and TRF) used three items in the construct. Internal reliabilities at pretest, 2 months, 4 months and 10 months were CBCL, α = 0.67, 0.72, 0.65, 0.69; YSR, α = 0.59, 0.64, 0.78, 0.76; TRF, α = 0.80, 0.82.

### 2.3. Interventions

#### 2.3.1. Methylphenidate Treatment

Short-acting methylphenidate was prescribed after consultation with a psychiatrist. Children received a prescription of three daily doses of methylphenidate (2.5 or 5 mg), 7 days a week. The psychiatrist called families weekly until optimal titration was obtained, and every four weeks, they met on location. If methylphenidate was not effective or side effects outweighed beneficial effects, the dose or medication type was changed, or the medication was stopped.

#### 2.3.2. Mindfulness Intervention MYmind

MYmind consisted of eight parallel sessions for children and their parents, and a booster session two months later. Each session took 1.5 h, and both children and parents were invited to practice meditation and yoga daily at home. Each week covered a different theme and different core mindfulness practices [60]. For details of the program, please see Table 1.

Treatment integrity was monitored by using the MYmind Treatment Adherence and Competence Scale (MYmind-TACS) [62]. In the MYmind children’s sessions, trainers delivered the exact exercises of the program in 85.8% of the cases and almost the exact exercises in 9.8% of the cases. For the MYmind parents’ sessions, this was 91.6% and 3.8%, respectively. Trainer’s competence was also rated with the MYmind-TACS, on a scale of 0–5: For children’s sessions, the average competence score was 4.29 (SD = 0.60); for parents’ sessions, average competence was 4.60 (SD = 0.27). Further details of the process of assessing treatment integrity are described in the study [48].

### 2.4. Analyses

Longitudinal multi-level analysis was conducted using SPSS 29. All respondents who participated in pretest surveys were included in our analysis. Drop-outs were asked to continue filling out surveys for the remaining three measures after the pretest. Little’s MCAR test found that data were missing completely at random. Missing data points were accounted for through maximum likelihood estimation (MLE) with an unstructured covariance structure for those who did not participate in subsequent surveys. MLE is a calculation that makes an educated estimate of what the missing data would have been, given available information. By using an unstructured covariance structure, no assumptions of correlations across time points can correlate freely with one another to better and more accurately reflect observations.

Pretest served as a reference category, and data were presented with both mindfulness and medication serving as reference categories. Only respondents who had completed the pretest were included in the analysis. Our protocol called for groups of 60 participants in each of the 2 treatment arms [58] rendering an expected power of 0.80. Ultimately, we had 51 and 47 participants in the treatment arms, giving a power of 0.79 for detecting a medium effect size (0.05) with a one-sided test.

## 3. Results

### 3.1. Outcome Domains

#### 3.1.1. Intervention Check

No differences were found in increases of mindful awareness between the children in the medication group versus those in the mindfulness group, at any time point. Please see Table 2 for all CAMM findings.

#### 3.1.2. Internalizing Problems

According to mothers, children’s Internalizing Problems showed a larger decrease in the medication group compared to the mindfulness group at 2 months (*p* < 0.05; ES = 0.46, almost medium effect), but from 4 months and beyond, there was no further difference between groups. Fathers, adolescents and teachers reported no differences between groups at any time points. For all the findings on Internalizing Problems, please see Table 3.

#### 3.1.3. Externalizing Problems

In line with Internalizing Problems, mothers’ reports of children’s Externalizing Problems showed a larger decrease in the medication group as compared to the mindfulness group at 2 months (*p* < 0.01; ES = 0.42, small to medium effect) and no difference at 4 or 10 months. Fathers reported a larger decrease in Externalizing Problems in the medication group at 2 months (*p* < 0.05; ES = 0.37, small effect) and 4 months (*p* < 0.05; ES = 0.35, small effect), but not at 10 months. No differences were reported between groups by children or teachers. For all findings on Externalizing Problems, please see Table 4.

#### 3.1.4. Social Problems

According to mothers and teachers, children’s social problems showed a larger decrease in the medication group at 2 months compared to the mindfulness group (*p* < 0.05; ES = 0.37, small effect and *p* < 0.05; ES = 0.42, small to medium effect, respectively). Fathers and children reported no differences between groups at any time point. For all findings on Social Problems, please see Table 5.

#### 3.1.5. Overall Results: Comorbidities over Time

At the short-term follow-up, for 42% of the outcome points (Internalizing Problems—mothers, Externalizing Problems—both parents, and Social Problems—mothers and teachers), results for children on medication improved more than for those following the family-mindfulness program. For 58% of the outcome points, results did not differ between groups, and for none of the outcomes did mindfulness outperform medication. For mid-term effects, on 11% of the outcome points (Externalizing Problems—fathers), effects were stronger in the medication group; for 89% of outcomes, results did not differ between treatment groups and for none of the outcomes did mindfulness outperform medication. For long-term effects, according to mothers, fathers, teachers and children, reductions in Internalizing, Externalizing and Social Problems did not differ between groups. Graphs of four informants by three outcome domains in two treatment groups are presented in the Supplementary Materials.

## 4. Discussion

In this study, we explored the short-, medium- and long-term effects of medication (methylphenidate for the child) versus family mindfulness (MYmind program for child and parents) on comorbid symptoms in children with ADHD. In line with our previous analysis focusing on the treatment effects of medication versus mindfulness on children’s core ADHD symptoms [48], we observed a similar pattern of results on these secondary symptoms; namely, medication was immediately effective and consistent across time, while effects of mindfulness became progressively more effective over time, but never outperformed those of medication. Thus, both treatment conditions have a similar effect on common comorbid symptoms as they do on core ADHD symptoms.

### 4.1. Internalizing Problems

Internalizing problems were reduced more in the children in the medication group as compared to those in the mindfulness group. However, this was only the case in the short term, according to mothers, not according to fathers, teachers and children. These findings were in line with previous studies that have found improvements in ADHD diagnosed children’s internalizing symptoms with both methylphenidate [63] and a mindfulness intervention [52].

### 4.2. Externalizing Problems

Externalizing problems were reduced more in the children in the medication group as compared to those in the mindfulness group in the short term, according to both parents and in the mid-term, according to fathers. According to teachers and the adolescents themselves, the long-term time points reduction in externalizing problems did not differ between groups. Thus, the pattern was similar to that for internalizing problems; medication had an immediate effect, and with time, there was less or no difference from mindfulness treatment anymore. There is evidence to suggest that medication can reduce impulsivity, which in turn has a positive impact on emotional regulation [64]. As methylphenidate has been associated with a reduction in emotional dysregulation [65], these reductions have been linked to improvements in some externalizing behaviors [40]. Similarly, mindfulness has been found to be linked to improvements in emotional regulation [66]. As we observed in a previous study of children with ADHD, mindfulness treatment has led to reductions in children’s externalizing behaviors as observed by mothers and fathers [52]. An important component of a family-based mindfulness intervention for children with ADHD is the mindfulness training of the parents, who not only model behavior for the child but also receive training to improve their parenting methods and their own emotional regulation. Assuming a reciprocal relationship between parenting behaviors (e.g., communication, involvement, discipline and punishment) and disruptive child behaviors, it makes sense to include the entire family system instead of just one or the other (e.g., [67]).

### 4.3. Social Problems

Social problems were reduced more in the children in the medication group as compared to those in the mindfulness group. However, this was only true at short-term assessment according to both mothers and teachers, not according to fathers or children, or at later points in time. In line with the other two domains, it seems that medication showed instant effects, where mindfulness caught up with time, but never “overtook” the effects of medication. This would indicate that children are immediately benefiting from medication and are interacting better with and are more accepted by their peers. This was also one of the conclusions of a systematic review about the short-term effects of methylphenidate on social impairments in children with ADHD [41].

### 4.4. Mechanisms

In the short term, 42% of the outcomes were in favor of the children taking medication; in the mid-term, this was the case for 11% of the outcomes, and in the long term, this dominant effect of medication seems to wane off and did not differ any longer from those in the mindfulness group. Although there were no moments where the improvements of children in the family-mindfulness program outperformed those in the medication group, for the (large) majority of outcomes, children in both groups improved, and reductions of internalizing, externalizing and social problems did not differ between the treatment conditions. Considering the drawbacks of medication, such as side effects, low adherence, dependency on an external agent instead of self-mastering long-term skills, mindfulness programs might offer an alternative. However, it is important to be aware that mindfulness is not a quick fix; it takes time, effort and some commitment, much more than taking medication.

Mothers reported immediate larger effects of medication than of mindfulness for children’s internalizing, externalizing and social problems. Assuming a traditional role pattern in the sense that mostly the mothers will be the ones spending more daytime hours with their children than fathers, they are more likely to notice the immediate positive effects of medication. Or perhaps this is related to the fact that more mothers participated in mindfulness than fathers. Overall, we see consistent improvements according to mothers at follow-ups at 4 and 10 months (fathers are more mixed). It could be that mothers have an adjusted relationship with the children that leads to altered expressions of the children’s presenting symptoms. Generally, medication is fast acting (e.g., [37]), whereas the parent/child dynamic takes time to alter.

### 4.5. Mindful Awareness

Surprisingly, although children in the mindfulness group showed improvements in mindful awareness and attention at mid-term, overall, children in the mindfulness program showed no larger improvement than those taking medication. Given that the mindfulness measure was intended as a treatment check, one might be tempted to interpret this as an initial failure of treatment. However, perhaps this lack of difference in improvements of mindfulness skills between the groups may be related to the choice of measurement; the CAMM assesses trait mindfulness. To develop mindfulness as an internal trait might be a longer-term process, and since the mindfulness program covers only a relatively short period of time, perhaps a measure of state mindfulness would have been more appropriate. This phenomenon of different understandings of one’s mindfulness post- versus pretest has been accounted for in recent research, which asks respondents not to judge post-test mindfulness on standard measures, but rather to judge themselves as to their levels “then” at pretest [68]. That is to say, “then” measures ask respondents to rate their post-test measures, given their conception of mindfulness at pretest as opposed to their newly founded understanding of mindfulness.

### 4.6. Long-Term Measures

Caution needs to be taken in drawing conclusions from the long-term measures. During the 6-month window between the 4-month and 10-month assessments, families were free to use other forms of treatment: Behavioral training, psychotherapy, parent training, and those allocated to medication could take part in mindfulness and vice versa. Just under half of the children in the mindfulness group started taking medication during this period, while nearly a quarter of the children allocated to the medication group stopped taking medication during the same time period, and around a third of the children in the medication group sought mindfulness-related contacts during this time period (see for details, ref. [48]).

### 4.7. Limitations

The lack of required adherence to the treatment protocol between these 4- and 10-month measurements is perhaps the largest methodological limitation of the study. Medication was already considered an evidence-based treatment for symptoms of ADHD and is part of most ADHD treatment guidelines (e.g., [69]), and it was therefore considered unethical by the external board to prevent families from the choice and option of taking medication. Seeing that children in the mindfulness group improved within 4 months on primary ADHD measures [48] as well as secondary measures of internalizing, externalizing and social problems, it might be reasonable to allow for future trials to require adherence to randomized protocols also for the longer term.

### 4.8. Future Studies and Clinical Implications

Future studies could further investigate the combined effect of mindfulness plus medication, and how, perhaps, mindfulness can help taper medication, as has been performed, for instance, in studies of Mindfulness-Based Cognitive Therapy and depression (e.g., [70]). The main clinical implication from this study is that a family-mindfulness intervention can be considered as a non-pharmacological alternative to medication for children with ADHD who are also experiencing internalizing, externalizing and social problems. Family MBIs could therefore be integrated more into mainstream clinical practice provided by psychologists or other trained mental health care professionals. As the evidence for effectiveness seems to rise rapidly, perhaps, the costs of these interventions could more often be covered by health care insurance.

## 5. Conclusions

Findings of this study can be summarized, along with five key points, as follows:

- Children with ADHD who take either medication or mindfulness treatment showed decreased internalizing, externalizing and social problems over time.
- At the short-term follow-up, reductions were larger for children in the medication group.
- At the medium-term follow-up, all but one of the comorbid symptom reductions did not differ between the children in the medication and the mindfulness groups.
- At the long-term follow-up, none of the four informants reported any differences between treatment groups in effects on internalizing, externalizing or social problems.
- Mindfulness training might be a viable option for families of children with ADHD who are seeking an alternative to medication to reduce comorbid externalizing, internalizing and/or social problems.

## Figures and Tables

**Table 1:** MYmind program: weekly session themes and core practices for children and parents.

	Core Practices MYmind	
	Children	Parents
Week 1. Beginners mind	Man from Mars; Breathing Meditation	Morning stress; Observe child with a beginner’s mind; Breathing meditation
Week 2. Home in your body	Bodyscan stretching; Ragdoll robot; Yoga	Bodyscan; Morning stress from perspective of a friend (compassion)
Week 3. The breath	Breathing space; Rubber duck	Breathing space: Meditation of body and breath
Week 4. Distractors	Meditation with distractors; Meditation on sounds	Sounds and thoughts meditation; Selfcare
Week 5. Stress	Halfway evaluation; Bodyscan only relaxing	Halfway evaluation; Choiceless awareness
Week 6. Highway, walkway	Breathing space on the highway; Walking meditation	Rupture and repair; Walking meditation
Week 7. Autonomy and acceptance	Quiz; Children as meditation and yoga teachers	Imagination exercise on acceptance and autonomy
Week 8. The future	Children lead meditation practices; Future plan	Parents follow their kids’ meditations; Future plan
Week 16. Each time starting anew	Bodyscan; Future mindfulness plan	Bodyscan; Review past 8 weeks; Future mindfulness plan

The Child Acceptance and Mindfulness Measure (CAMM) was used as an intervention check. Utilizing 25 items, the CAMM [61] measures children’s mindfulness and their capacity to observe internal experience, act with awareness and non-judgmentally accept internal experiences. The CAMM was only filled out by children who were 11 years or older. Example items from the CAMM are “I tell myself that I shouldn’t feel the way I am feeling” and “I push away thoughts I don’t like”. Internal reliabilities at pretest, 2 months, 4 months, and 10 months were α = 0.77, 0.73, 0.75 and 0.84.

**Table 2:** Standardized parameter estimates per reporter and per treatment group, at 2-month, 4-month and 10-month follow-up on Child Acceptance and Mindfulness Measure (CAMM).

	CAMM
	PE (SE)
2-mo. follow-up (vs. pretest) mindfulness	0.15 (0.23)
4-mo. follow-up (vs. pretest) mindfulness	0.62 (0.28) *
10-mo. follow-up (vs. pretest) mindfulness	0.56 (0.28)
2-mo. follow-up (vs. pretest) medication	0.24 (0.23)
4-mo. follow-up (vs. pretest) medication	0.38 (0.29)
10-mo. follow-up (vs. pretest) medication	0.30 (0.30)
Medication–mindfulness difference at pretest	−0.45 (0.31)
Medication–mindfulness pretest to 2 months	−0.09 (0.32)
Medication–mindfulness pretest to 4 months	0.24 (0.40)
Medication–mindfulness pretest to 10 months	0.26 (0.41)

Notes. Parameter estimates can be interpreted as Cohen’s d effect size as an indication of clinical significance. * *p* < 0.05.

**Table 3:** Standardized parameter estimates per reporter and per treatment group, at 2-month, 4-month and 10-month follow-up on ASEBA internalizing problems.

	ASEBA Internalizing Problems			
	Mothers	Fathers	Adolescents	Teachers
	PE (SE)	PE (SE)	PE (SE)	PE (SE)
2-mo. follow-up (vs. pretest) mindfulness	−0.22 (0.13)	−0.29 (0.12) *	−0.18 (0.18)	−0.28 (0.09) **
4-mo. follow-up (vs. pretest) mindfulness	−0.36 (0.12) **	−0.36 (0.14) *	−0.40 (0.23)	^a^
10-mo. follow-up (vs. pretest) mindfulness	−0.43 (0.13) **	−0.31 (0.16)	−0.22 (0.22)	^a^
2-mo. follow-up (vs. pretest) medication	−0.69 (0.13) ***	−0.33 (0.13) *	−0.52 (0.18) **	−0.35 (0.08) ***
4-mo. follow-up (vs. pretest) medication	−0.63 (0.13) ***	−0.60 (0.14) ***	−0.72 (0.24) **	^a^
10-mo. follow-up (vs. pretest) medication	−0.52 (0.13) ***	−0.73 (0.17) ***	−0.79 (0.24) **	^a^
Medication–mindfulness difference at pretest	−0.25 (0.21)	−0.20 (0.23)	0.08 (0.29)	−0.10 (0.19)
Medication–mindfulness pretest to 2 months	0.46 (0.18) *	0.04 (0.17)	0.34 (0.25)	0.07 (0.12)
Medication–mindfulness pretest to 4 months	0.27 (0.17)	0.25 (0.20)	0.32 (0.34)	^a^
Medication–mindfulness	0.10 (0.18)	0.42 (0.24)	0.57 (0.33)	^a^
pretest to 10 months				

Notes. Parameter estimates can be interpreted as Cohen’s d effect size as an indication of clinical significance. Negative estimates indicate a reduction in symptoms at follow-ups. * *p* < 0.05, ** *p* < 0.01, *** *p* < 0.001, ^a^ = not measured.

**Table 4:** Standardized parameter estimates per reporter and per treatment group, at 2-month, 4-month and 10-month follow-up on ASEBA externalizing problems.

	ASEBA Externalizing Problems			
	Mothers	Fathers	Adolescents	Teachers
	PE (SE)	PE (SE)	PE (SE)	PE (SE)
2-mo. follow-up (vs. pretest) mindfulness	−0.17 (0.11)	−0.15 (0.11)	−0.30 (0.18)	−0.09 (0.07)
4-mo. follow-up (vs. pretest) mindfulness	−0.39 (0.11) ***	−0.17 (0.11)	−0.62 (0.22) **	^a^
10-mo. follow-up (vs. pretest) mindfulness	−0.42 (0.10) ***	−0.35 (0.16) *	−0.25 (0.27)	^a^
2-mo. follow-up (vs. pretest) medication	−0.60 (0.11) ***	−0.51 (0.12) ***	−0.37 (0.18) *	−0.25 (0.07) ***
4-mo. follow-up (vs. pretest) medication	−0.57 (0.11) ***	−0.52 (0.11) ***	−0.47 (0.23) *	^a^
10-mo. follow-up (vs. pretest) medication	−0.55 (0.11) ***	−0.70 (0.17) ***	−0.68 (0.28) *	^a^
Medication—mindfulness difference at pretest	−0.24 (0.20)	−0.16 (0.22)	−0.04 (0.26)	−0.02 (0.19)
Medication—mindfulness	0.42 (0.16) **	0.37 (0.16) *	0.07 (0.26)	0.17 (0.10)
pretest to 2 months				
Medication—mindfulness	0.18 (0.15)	0.35 (0.16) *	−0.15 (0.32)	^a^
pretest to 4 months				
Medication—mindfulness	0.13 (0.15)	0.35 (0.23)	0.43 (0.39)	^a^
pretest to 10 months				

Notes. Parameter estimates can be interpreted as Cohen’s d effect size as an indication of clinical significance. Negative estimates indicate a reduction in symptoms at follow-ups. * *p* < 0.05, ** *p* < 0.01, *** *p* < 0.001, ^a^ = not measured.

**Table 5:** Standardized parameter estimates per reporter and per treatment group, at 2-month, 4-month and 10-month follow-up on ASEBA social problems.

	ASEBA Social Problems			
	Mothers	Fathers	Adolescents	Teachers
	PE (SE)	PE (SE)	PE (SE)	PE (SE)
2-mo. follow-up (vs. pretest) mindfulness	−0.20 (0.12)	−0.21 (0.14)	−0.39 (0.19) *	0.03 (0.13)
4-mo. follow-up (vs. pretest) mindfulness	−0.41 (0.12) ***	−0.19 (0.12)	−0.63 (0.23) **	^a^
10-mo. follow-up (vs. pretest) mindfulness	−0.36 (0.15) *	−0.32 (0.14) *	−0.36 (0.24)	^a^
2-mo. follow-up (vs. pretest) medication	−0.57 (0.12) ***	−0.23 (0.14)	−0.17 (0.19)	−0.39 (0.13) **
4-mo. follow-up (vs. pretest) medication	−0.37 (0.12) **	−0.44 (0.12) ***	−0.14 (0.23)	^a^
10-mo. follow-up (vs. pretest) medication	−0.49 (0.16) **	−0.63 (0.14) ***	−0.66 (0.25) *	^a^
Medication–mindfulness difference at pretest	−0.14 (0.21)	−0.03 (0.24)	0.40 (0.31)	0.09 (0.26)
Medication–mindfulness	0.37 (0.17) *	0.01 (0.20)	−0.23 (0.26)	0.42 (0.18) *
pretest to 2 months				
Medication–mindfulness	−0.04 (0.17)	0.25 (0.17)	−0.48 (0.33)	^a^
pretest to 4 months				
Medication–mindfulness	0.14 (0.22)	0.30 (0.20)	0.30 (0.35)	^a^
pretest to 10 months				

Notes. Parameter estimates can be interpreted as Cohen’s d effect size as an indication of clinical significance. Negative estimates indicate a reduction in symptoms at follow-ups., * *p* < 0.05, ** *p* < 0.01, *** *p* < 0.001, ^a^ = not measured.

## Data Availability

Data from this study are stored in the University’s repository.

## Supplementary Materials

The following supporting information can be downloaded at: https://www.mdpi.com/article/10.3390/children12060681/s1, Table S1. Means and Standard Deviations for Children’s self-reported secondary outcomes in the MYmind Mindfulness-Based Intervention and Medication Group on ASEBA Internalizing Problems, ASEBA Externalizing Problems and ASEBA Social Problems and the Child Acceptance and Mindfulness Measure (CAMM); Table S2. Means and Standard Deviations for Children’s self-reported secondary outcomes in the MYmind Mindfulness-Based Intervention and Medication Group on ASEBA Internalizing Anxiety/Depression Problems, ASEBA Internalizing Withdrawn/Depressed Problems, ASEBA Internalizing Somatic Problems, ASEBA Externalizing Rule Breaking Behavior Problems and ASEBA Externalizing Aggressive Behavior Problems; Table S3. Standardized Parameter Estimates per Reporter and per Treatment Group, at 2-month-, 4-month- and 10-month follow-up on ASEBA Internalizing Anxiety/Depression Problems; Table S4. Standardized Parameter Estimates per Reporter and per Treatment Group, at 2-month-, 4-month- and 10-month follow-up on ASEBA Internalizing Withdrawn/Depressed Problems; Table S5. Standardized Parameter Estimates per Reporter and per Treatment Group, at 2-month-, 4-month- and 10-month follow-up on ASEBA Internalizing Somatic Problems; Table S6. Standardized Parameter Estimates per Reporter and per Treatment Group, at 2-month-, 4-month- and 10-month follow-up on ASEBA Externalizing Rule Breaking Behavior Problems; Table S7. Standardized Parameter Estimates per Reporter and per Treatment Group, at 2-month-, 4-month- and 10-month follow-up on ASEBA Externalizing Aggressive Behavior Problems; Figure S1. Line graphs of Z-scores for two groups—Mindfulness (MFN) and Medication (MED)—across the following variables: (A) ASEBA Internalizing Problems-Mothers, (B) ASEBA Externalizing Problems-Mothers, (C) ASEBA Social Problems-Mothers, (D) ASEBA Internalizing Problems-Fathers, (E) ASEBA Externalizing Problems-Fathers, (F) ASEBA Social Problems-Fathers, (G) ASEBA Internalizing Problems-Adolescents, (H) ASEBA Externalizing Problems-Adolescents, (I) ASEBA Social Problems-Adolescents, (J) ASEBA Internalizing Problems-Teachers, (K) ASEBA Externalizing Problems-Teachers and (L) ASEBA Social Problems-Teachers.
