# Supplementary material for: Methylphenidate or Family Mindfulness? Effects on Internalizing, Externalizing and Social Problems in Children with Attention Deficit Hyperactivity Disorder
Source: Children (Basel). 2025 May 26;12(6):681. doi: 10.3390/children12060681 (PMC12190967; doi:10.3390/children12060681)
Supplement: Supplementary file 1 [file children-12-00681-s001.zip › children-3619912-supplementary.pdf]

Table S1. Means and Standard Deviations for Children's self-reported secondary outcomes in the MYmind Mindfulness Based Intervention and Medication Group on ASEBA Internalizing Problems, ASEBA Externalizing Problems, ASEBA Social Problems and Child Acceptance and Mindfulness Measure (CAMM).

	Pre-Test			2-Months			4-Months			10-Months		
	M	*SD*	*N*	*M*	*SD*	*N*	*M*	*SD*	*N*	*M*	*SD*	*N*
**ASEBA Internalizing Problems**												
Mindfulness Mothers	0.33	1.07	46	0.09	0.94	42	0.01	0.99	43	0.02	0.94	38
Medication Mothers	0.58	1.01	45	-0.16	1.05	39	-0.15	1.03	39	0.00	1.13	35
Mindfulness Fathers	0.12	1.10	42	-0.24	0.87	40	-0.24	1.05	37	-0.22	0.92	34
Medication Fathers	0.30	0.92	38	-0.04	0.97	34	-0.28	0.93	36	-0.32	0.84	29
Mindfulness Child	0.19	0.86	19	-0.02	1.03	18	-0.29	1.09	16	0.10	1.11	16
Medication Child	0.09	1.00	21	-0.31	0.98	17	-0.66	1.17	14	-0.70	1.12	13
Mindfulness Teacher	0.22	0.77	39	-0.06	0.86	39	-	-	-	-	-	-
Medication Teacher	0.32	0.93	42	-0.03	0.95	42	-	-	-	-	-	-
**ASEBA Externalizing Problems**												
Mindfulness Mothers	0.27	1.03	46	0.09	0.94	42	-0.11	0.97	43	-0.10	1.00	38
Medication Mothers	0.50	0.96	45	-0.13	1.08	39	-0.21	0.97	39	-0.13	0.87	35
Mindfulness Fathers	0.12	1.08	42	-0.05	1.08	40	-0.11	1.12	37	-0.20	1.01	34
Medication Fathers	0.29	0.85	38	-0.31	0.90	34	-0.26	0.95	36	-0.41	0.92	29
Mindfulness Child	0.07	0.71	19	-0.24	0.85	18	-0.61	1.11	16	-0.15	1.22	16
Medication Child	0.11	0.96	21	-0.29	1.04	17	-0.46	1.23	14	-0.69	0.95	13
Mindfulness Teacher	0.38	0.85	39	0.29	0.85	39	-	-	-	-	-	-
Medication Teacher	0.40	0.91	42	0.15	0.95	42	-	-	-	-	-	-
**ASEBA Social Problems**												
Mindfulness Mothers	0.17	1.14	46	-0.06	0.92	42	-0.17	0.70	43	-0.12	0.97	38
Medication Mothers	0.34	0.89	45	-0.27	0.86	39	-0.08	1.01	39	-0.19	0.90	35
Mindfulness Fathers	0.20	1.13	42	-0.02	1.00	40	-0.06	1.01	37	-0.10	0.88	34
Medication Fathers	0.21	0.98	38	-0.11	0.98	34	-0.25	0.82	36	-0.41	0.64	29
Mindfulness Child	0.55	0.97	19	0.16	1.19	18	-0.14	0.94	16	0.19	1.13	16
Medication Child	0.14	1.04	21	0.06	1.00	17	0.09	1.20	14	-0.40	0.94	13
Mindfulness Teacher	0.22	1.15	39	0.24	1.23	39	-	-	-	-	-	-
Medication Teacher	0.13	1.18	42	-0.26	0.86	42	-	-	-	-	-	-
**Child Acceptance and Mindfulness Measure (CAMM)**												

Mindfulness Child	-0.50	1.10	19	-0.29	1.17	18	0.15	0.96	16	0.03	1.10	16
Medication Child	-0.04	0.92	21	0.18	0.70	17	0.31	0.93	14	0.40	0.88	13

Table S2. Means and Standard Deviations for Children's self-reported secondary outcomes in the MYmind Mindfulness Based Intervention and Medication Group on ASEBA Internalizing Anxiety/Depression Problems, ASEBA Internalizing Withdrawn/Depressed Problems, ASEBA Internalizing Somatic Problems, ASEBA Externalizing Rule Breaking Behavior Problems and ASEBA Externalizing Aggressive Behavior Problems. .

	Pre-Test			2-Months			4-Months			10-Months		
	M	*SD*	*N*	*M*	*SD*	*N*	*M*	*SD*	*N*	*M*	*SD*	*N*
**ASEBA Internalizing Anxiety/Depression Problems**												
Mindfulness Mothers	0.17	1.14	47	-0.06	0.93	43	-0.14	0.91	44	-0.19	0.97	39
Medication Mothers	0.51	1.13	45	-0.04	1.06	39	-0.21	0.80	39	0.15	1.28	35
Mindfulness Fathers	0.04	1.00	41	-0.26	0.70	39	-0.25	0.86	36	-0.21	0.99	33
Medication Fathers	0.27	1.15	38	-0.09	0.94	34	-0.26	0.73	36	-0.42	0.51	29
Mindfulness Child	0.09	0.90	19	-0.04	0.79	18	-0.18	1.02	16	0.35	1.23	16
Medication Child	0.31	1.22	21	-0.12	1.00	17	-0.14	0.98	14	-0.21	0.94	13
Mindfulness Teacher	0.13	0.84	39	-0.08	0.84	39	-	-	-	-	-	-
Medication Teacher	0.43	1.25	42	0.11	1.05	42	-	-	-	-	-	-
**ASEBA Internalizing Withdrawn/Depressed Problems**												
Mindfulness Mothers	0.38	1.36	47	0.01	0.92	43	0.16	0.97	44	0.17	1.01	39
Medication Mothers	0.45	1.28	45	-0.18	0.97	39	-0.05	0.92	39	0.24	1.55	35
Mindfulness Fathers	0.28	1.22	41	-0.09	0.91	39	0.04	0.96	36	0.10	1.04	33
Medication Fathers	0.12	0.89	38	0.01	0.96	34	-0.15	0.90	36	-0.28	0.78	29
Mindfulness Child	-0.14	0.87	19	-0.18	0.90	18	-0.20	0.80	16	-0.03	0.79	16
Medication Child	-0.35	0.61	21	-0.70	0.30	17	-0.57	0.48	14	-0.43	0.77	13
Mindfulness Teacher	0.01	0.91	39	-0.16	0.80	39	-	-	-	-	-	-
Medication Teacher	-0.01	0.85	42	-0.18	0.79	42	-	-	-	-	-	-
**ASEBA Internalizing Somatic Problems**												
Mindfulness Mothers	0.54	1.21	47	0.23	1.10	43	0.15	0.99	44	0.07	0.98	39
Medication Mothers	0.63	1.11	45	-0.15	1.02	39	0.09	1.12	39	-0.03	1.03	35
Mindfulness Fathers	0.09	1.01	41	-0.24	0.78	39	-0.19	0.83	36	-0.31	0.66	33
Medication Fathers	0.23	1.09	38	0.02	0.79	34	-0.26	0.72	36	-0.18	0.88	29
Mindfulness Child	0.43	1.19	19	0.18	1.26	18	0.01	1.04	16	0.17	1.11	16
Medication Child	0.27	1.18	21	0.17	1.01	17	-0.31	0.82	14	-0.37	0.86	13

Mindfulness Teacher	-0.34	0.89	39	-0.43	0.89	39	-	-	-	-	-	-
Medication Teacher	-0.23	0.86	42	-0.37	0.60	42	-	-	-	-	-	-
**ASEBA Externalizing Rule Breaking Behavior Problems**												
Mindfulness Mothers	0.28	1.04	47	-0.23	0.79	43	-0.31	0.70	44	-0.18	1.04	39
Medication Mothers	0.62	1.21	45	-0.03	1.01	39	-0.13	0.79	39	-0.22	0.74	35
Mindfulness Fathers	0.27	1.13	41	-0.17	0.93	39	-0.10	1.02	36	-0.22	0.90	33
Medication Fathers	0.36	1.11	38	-0.26	0.75	34	-0.13	0.87	36	-0.20	0.92	29
Mindfulness Child	0.06	0.77	19	-0.18	0.93	18	-0.30	0.89	16	0.32	1.41	16
Medication Child	0.09	1.00	21	-0.13	1.07	17	-0.14	0.84	14	-0.23	0.97	13
Mindfulness Teacher	0.01	0.95	39	-0.03	0.96	39	-	-	-	-	-	-
Medication Teacher	0.36	1.20	42	0.17	1.17	42	-	-	-	-	-	-
**ASEBA Externalizing Aggressive Behavior Problems**												
Mindfulness Mothers	0.27	1.05	47	0.12	1.08	43	-0.05	0.79	44	-0.06	0.98	39
Medication Mothers	0.41	1.12	45	-0.08	1.01	39	-0.18	0.86	39	-0.19	0.74	35
Mindfulness Fathers	0.14	1.27	41	0.09	1.22	39	0.02	1.07	36	-0.10	0.89	33
Medication Fathers	0.07	0.96	38	-0.32	0.76	34	-0.29	0.74	36	-0.45	0.52	29
Mindfulness Child	-0.12	0.71	19	-0.33	0.74	18	-0.37	0.87	16	-0.16	1.17	16
Medication Child	0.08	1.11	21	-0.23	0.89	17	-0.18	1.02	14	-0.59	0.65	13
Mindfulness Teacher	0.36	1.00	39	0.26	0.98	39	-	-	-	-	-	-
Medication Teacher	0.29	1.15	42	0.07	1.15	42	-	-	-	-	-	-

Table S3. Standardized Parameter Estimates per Reporter and per Treatment Group, at 2-months-, 4-months- and 10-months follow-up on ASEBA Internalizing Anxiety/Depression Problems.

	**ASEBA Internalizing Anxiety/Depression Problems**			
	Mothers	Fathers	Adolescents	Teachers
	PE (*SE*)	*PE* (*SE*)	*PE* (*SE*)	*PE* (*SE*)
2-mo. follow-up (vs. pretest) Mindfulness	-0.22 (0.13)	-0.27 (0.14) *	-0.14 (0.20)	-0.21 (0.10) *
4-mo. follow-up (vs. pretest) Mindfulness	-0.35 (0.14) *	-0.25 (0.14)	-0.18 (0.21)	[a]
10-mo. follow-up (vs. pretest) Mindfulness	-0.45 (0.17) **	-0.20 (0.16)	0.16 (0.26)	[a]
2-mo. follow-up (vs. pretest) Medication	-0.57 (0.14) ***	-0.37 (0.14) *	-0.47 (0.20) *	-0.32 (0.10) **
4-mo. follow-up (vs. pretest) Medication	-0.65 (0.15) ***	-0.54 (0.14) ***	-0.39 (0.21)	[a]
10-mo. follow-up (vs. pretest) Medication	-0.40 (0.17) *	-0.76 (0.16) ***	-0.52 (0.27)	[a]
Medication - Mindfulness difference at pretest	-0.34 (0.23)	-0.23 (0.24)	-0.25 (0.33)	-0.30 (0.24)
Medication - Mindfulness pretest to 2-mo.	0.35 (0.19)	0.09 (0.20)	0.34 (0.28)	0.12 (0.14)
Medication - Mindfulness pretest to 4-mo.	0.30 (0.20)	0.29 (0.20)	0.20 (0.29)	[a]
Medication - Mindfulness pretest to 10-mo.	-0.05 (0.24)	0.56 (0.23) *	0.68 (0.37)	[a]

Notes. Parameter Estimates can be interpreted as Cohen's *d* effect size. Negative estimates indicate reduction in symptoms at follow-ups. + $p < 0.10$, * $p < 0.05$, ** $p < 0.01$, *** $p < 0.001$, [a] = not measured.

Table S4. Standardized Parameter Estimates per Reporter and per Treatment Group, at 2-months-, 4-months- and 10-months follow-up on ASEBA Internalizing Withdrawn/Depressed Problems.

	**ASEBA Internalizing Withdrawn/Depressed Problems**			
	Mothers	Fathers	Adolescents	Teachers
	PE (*SE*)	*PE* (*SE*)	*PE* (*SE*)	*PE* (*SE*)
2-mo. follow-up (vs. pretest) Mindfulness	-0.33 (0.17)	-0.33 (0.15) *	-0.04 (0.19)	-0.17 (0.09)
4-mo. follow-up (vs. pretest) Mindfulness	-0.26 (0.16)	-0.20 (0.14)	-0.09 (0.20)	a
10-mo. follow-up (vs. pretest) Mindfulness	-0.33 (0.18)	-0.12 (0.17)	0.07 (0.20)	a
2-mo. follow-up (vs. pretest) Medication	-0.56 (0.17) **	-0.09 (0.16)	-0.36 (0.19)	-0.17 (0.09) *
4-mo. follow-up (vs. pretest) Medication	-0.42 (0.16) *	-0.28 (0.15)	-0.22 (0.20)	a
10-mo. follow-up (vs. pretest) Medication	-0.18 (0.18)	-0.46 (0.18) *	-0.09 (0.21)	a
Medication - Mindfulness difference at pretest	-0.07 (0.27)	0.16 (0.24)	0.22 (0.23)	0.02 (0.19)
Medication - Mindfulness pretest to 2-mo.	0.23 (0.24)	-0.25 (0.22)	0.32 (0.26)	0.00 (0.12)
Medication - Mindfulness pretest to 4-mo.	0.30 (0.20)	0.29 (0.20)	0.20 (0.29)	a
Medication - Mindfulness pretest to 10-mo.	0.16 (0.22)	0.08 (0.21)	0.13 (0.28)	a

Notes. Parameter Estimates can be interpreted as Cohen's *d* effect size. Negative estimates indicate reduction in symptoms at follow-ups.
+ $p < 0.10$, * $p < 0.05$, ** $p < 0.01$, *** $p < 0.001$, [a] = not measured.

Table S5. Standardized Parameter Estimates per Reporter and per Treatment Group, at 2-months-, 4-months- and 10-months follow-up on ASEBA Internalizing Somatic Problems.

	**ASEBA Internalizing Somatic Problems**			
	Mothers	Fathers	Adolescents	Teachers
	PE (*SE*)	*PE* (*SE*)	*PE* (*SE*)	*PE* (*SE*)
2-mo. follow-up (vs. pretest) Mindfulness	-0.29 (0.14) *	-0.30 (0.15) *	-0.23 (0.25)	-0.09 (0.11)
4-mo. follow-up (vs. pretest) Mindfulness	-0.40 (0.15) **	-0.27 (0.16)	-0.35 (0.24)	a
10-mo. follow-up (vs. pretest) Mindfulness	-0.56 (0.15)	-0.37 (0.18) *	-0.31 (0.29)	a
2-mo. follow-up (vs. pretest) Medication	-0.73 (0.15) ***	-0.20 (0.16)	-0.16 (0.24)	-0.14 (0.10)
4-mo. follow-up (vs. pretest) Medication	-0.49 (0.15) **	-0.49 (0.16) **	-0.58 (0.25) *	a
10-mo. follow-up (vs. pretest) Medication	-0.58 (0.16) ***	-0.45 (0.19) *	-0.55 (0.29)	a
Medication - Mindfulness difference at pretest	-0.09 (0.24)	-0.14 (0.23)	0.13 (0.36)	-0.12 (0.19)
Medication - Mindfulness pretest to 2-mo.	0.44 (0.20) *	-0.10 (0.22)	-0.07 (0.35)	0.05 (0.15)
Medication - Mindfulness pretest to 4-mo.	0.30 (0.20)	0.29 (0.20)	0.20 (0.29)	a

Medication - Mindfulness pretest to 10-mo.	0.09 (0.21)	0.22 (0.23)	0.23 (0.35)	a

Notes. Parameter Estimates can be interpreted as Cohen's *d* effect size. Negative estimates indicate reduction in symptoms at follow-ups.
+ $p < 0.10$, * $p < 0.05$, ** $p < 0.01$, *** $p < 0.001$, a = not measured.

Table S6. Standardized Parameter Estimates per Reporter and per Treatment Group, at 2-months-, 4-months- and 10-months follow-up on ASEBA Externalizing Rule Breaking Behavior Problems.

	**ASEBA Externalizing Rule Breaking Behavior Problems**			
	Mothers	Fathers	Adolescents	Teachers
	PE (*SE*)	*PE* (*SE*)	*PE* (*SE*)	*PE* (*SE*)
2-mo. follow-up (vs. pretest) Mindfulness	-0.49 (0.13) ***	-0.43 (0.12) ***	-0.19 (0.20)	-0.03 (0.10)
4-mo. follow-up (vs. pretest) Mindfulness	-0.58 (0.13) ***	-0.32 (0.14) *	-0.28 (0.22)	a
10-mo. follow-up (vs. pretest) Mindfulness	-0.50 (0.14) ***	-0.47 (0.17) **	0.26 (0.30)	a
2-mo. follow-up (vs. pretest) Medication	-0.63 (0.14) ***	-0.52 (0.13) ***	-0.13 (0.20)	-0.19 (0.10)
4-mo. follow-up (vs. pretest) Medication	-0.64 (0.13) ***	-0.48 (0.14) ***	-0.18 (0.22)	a
10-mo. follow-up (vs. pretest) Medication	-0.66 (0.15) ***	-0.56 (0.17) **	-0.14 (0.32)	a
Medication - Mindfulness difference at pretest	-0.34 (0.23)	-0.08 (0.25)	-0.06 (0.28)	-0.35 (0.24)
Medication - Mindfulness pretest to 2-mo.	0.14 (0.19)	0.08 (0.18)	-0.06 (0.29)	0.16 (0.14)
Medication - Mindfulness pretest to 4-mo.	0.30 (0.20)	0.29 (0.20)	0.20 (0.29)	a
Medication - Mindfulness pretest to 10-mo.	0.06 (0.19)	0.16 (0.19)	-0.10 (0.31)	a

Notes. Parameter Estimates can be interpreted as Cohen's *d* effect size. Negative estimates indicate reduction in symptoms at follow-ups.
+ $p < 0.10$, * $p < 0.05$, ** $p < 0.01$, *** $p < 0.001$, a = not measured.

Table S7. Standardized Parameter Estimates per Reporter and per Treatment Group, at 2-months-, 4-months- and 10-months follow-up on ASEBA Externalizing Aggressive Behavior Problems.

	**ASEBA Externalizing Aggressive Behavior Problems**			
	Mothers	Fathers	Adolescents	Teachers
	PE (*SE*)	*PE* (*SE*)	*PE* (*SE*)	*PE* (*SE*)
2-mo. follow-up (vs. pretest) Mindfulness	-0.14 (0.12)	-0.07 (0.11)	-0.23 (0.21)	-0.10 (0.08)
4-mo. follow-up (vs. pretest) Mindfulness	-0.33 (0.10) **	-0.04 (0.12)	-0.21 (0.22)	a
10-mo. follow-up (vs. pretest) Mindfulness	-0.39 (0.11) ***	-0.23 (0.15)	-0.07 (0.32)	a
2-mo. follow-up (vs. pretest) Medication	-0.44 (0.12) ***	-0.31 (0.12) *	-0.34 (0.20)	-0.21 (0.08) **
4-mo. follow-up (vs. pretest) Medication	-0.48 (0.10) ***	-0.32 (0.12) **	-0.29 (0.23)	a
10-mo. follow-up (vs. pretest) Medication	-0.49 (0.12) ***	-0.52 (0.16) **	-0.72 (0.33) *	a
Medication - Mindfulness difference at pretest	-0.15 (0.22)	0.08 (0.25)	-0.19 (0.29)	0.08 (0.24)
Medication - Mindfulness pretest to 2-mo.	0.30 (0.17)	0.24 (0.16)	0.12 (0.29)	0.11 (0.11)

Medication - Mindfulness pretest to 4-mo.	0.30 (0.20)	0.29 (0.20)	0.20 (0.29)	[a]
Medication - Mindfulness pretest to 10-mo.	0.14 (0.14)	0.28 (0.16)	0.08 (0.32)	[a]

Notes. Parameter Estimates can be interpreted as Cohen's *d* effect size. Negative estimates indicate reduction in symptoms at follow-ups.
[+] $p < 0.10$, * $p < 0.05$, ** $p < 0.01$, *** $p < 0.001$, [a] = not measured.

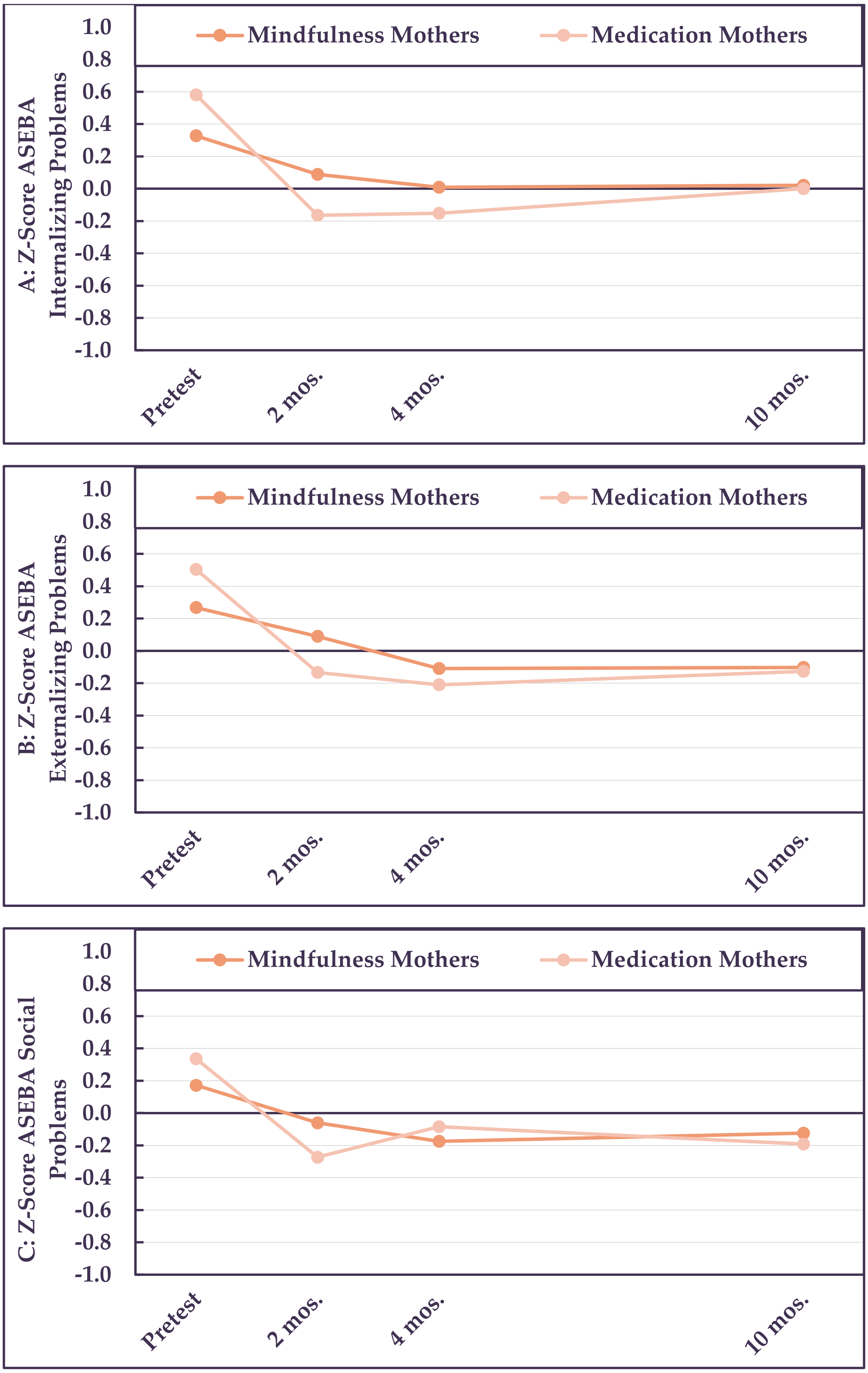
Mindfulness Mothers
Medication Mothers
A: Z-Score ASEBA Internalizing Problems
B: Z-Score ASEBA Externalizing Problems
C: Z-Score ASEBA Social Problems
1.0
0.8
0.6
0.4
0.2
0.0
-0.2
-0.4
-0.6
-0.8
-1.0
Pretest
2 mos.
4 mos.
10 mos.

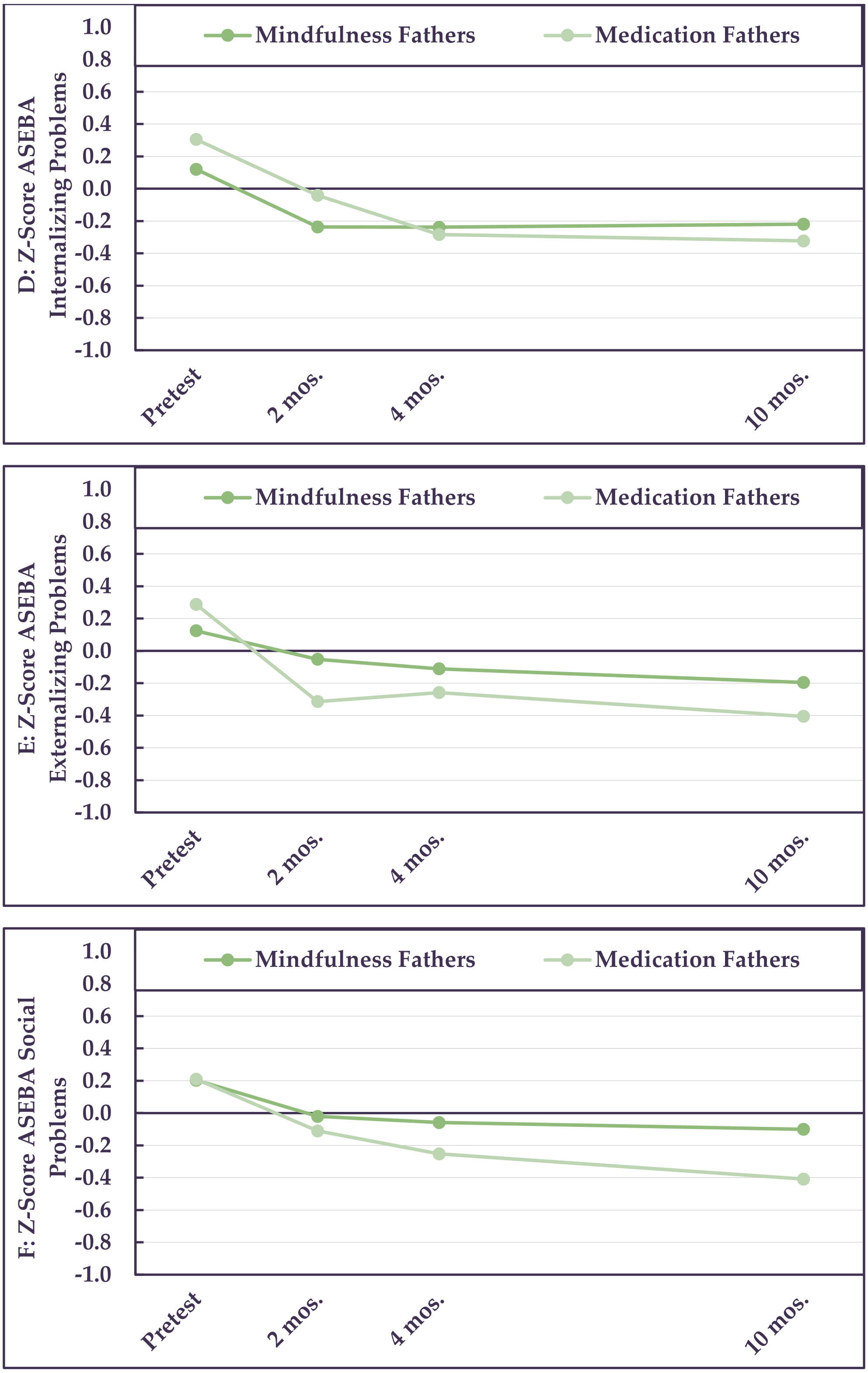
Mindfulness Fathers
Medication Fathers
D: Z-Score ASEBA Internalizing Problems
1.0
0.8
0.6
0.4
0.2
0.0
-0.2
-0.4
-0.6
-0.8
-1.0
Pretest
2 mos.
4 mos.
10 mos.
Mindfulness Fathers
Medication Fathers
E: Z-Score ASEBA Externalizing Problems
1.0
0.8
0.6
0.4
0.2
0.0
-0.2
-0.4
-0.6
-0.8
-1.0
Pretest
2 mos.
4 mos.
10 mos.
Mindfulness Fathers
Medication Fathers
F: Z-Score ASEBA Social Problems
1.0
0.8
0.6
0.4
0.2
0.0
-0.2
-0.4
-0.6
-0.8
-1.0
Pretest
2 mos.
4 mos.
10 mos.

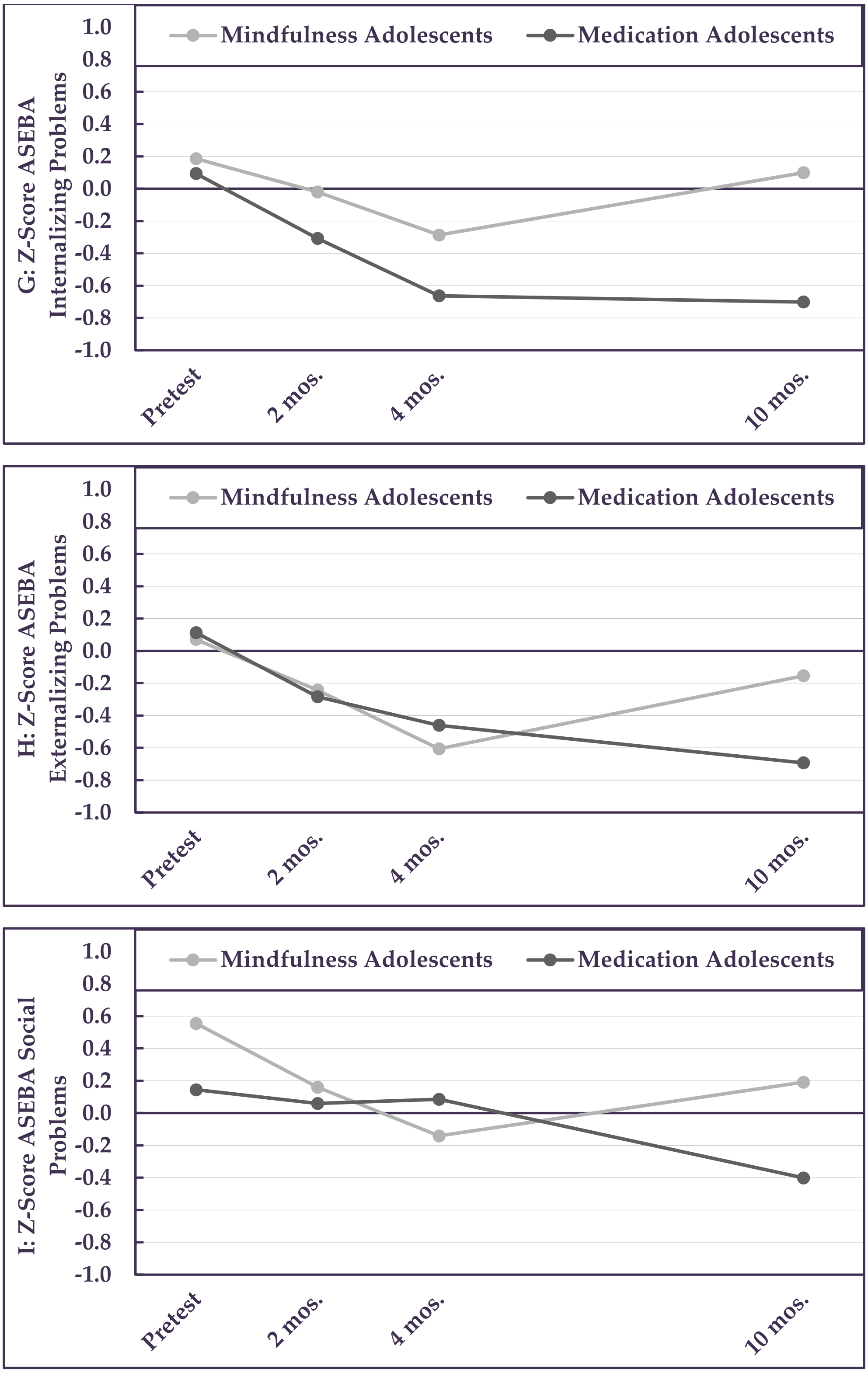
Mindfulness Adolescents
Medication Adolescents
G: Z-Score ASEBA Internalizing Problems
H: Z-Score ASEBA Externalizing Problems
I: Z-Score ASEBA Social Problems
1.0
0.8
0.6
0.4
0.2
0.0
-0.2
-0.4
-0.6
-0.8
-1.0
Pretest
2 mos.
4 mos.
10 mos.

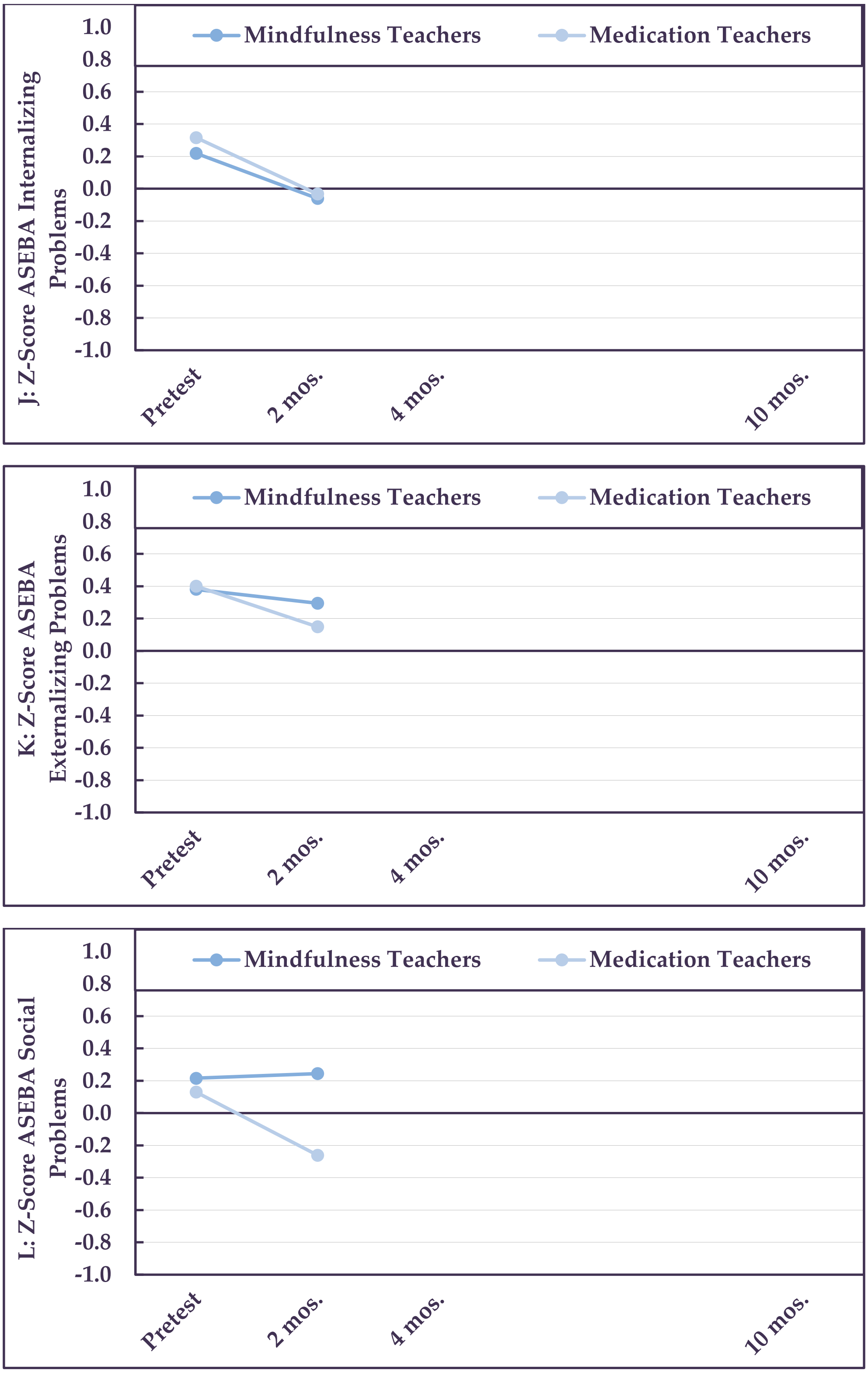

Figure S1. Line graphs of Z-scores for two groups—Mindfulness (MFN) and Medication (MED)—across the following variables: (A) ASEBA Internalizing Problems-Mothers, (B) ASEBA Externalizing Problems-Mothers, (C) ASEBA Social Problems-Mothers, (D) ASEBA Internalizing Problems-Fathers, (E) ASEBA Externalizing Problems-Fathers, (F) ASEBA Social Problems-Fathers, (G) ASEBA Internalizing Problems-Adolescents, (H) ASEBA Externalizing Problems-Adolescents, (I) ASEBA Social Problems-Adolescents, (J) ASEBA Internalizing Problems-Teachers, (K) ASEBA Externalizing Problems-Teachers and (L) ASEBA Social Problems-Teachers. Each panel (A–L) represents the standardized scores for the respective variable, with separate lines for Mindfulness and Medication groups.
